# 

**Published:** 1874-02

**Authors:** 


### CONTENTS:

### ORIGINAL COMMUNICATIONS.

ARTICLE I.—The Larynx the Source of the Vowel Sounds. (Concluded.) By Thomas Brian Gunning, D. D. S., M. D. .... 433

ARTICLE II.—Filling Cavities of Decay in Proximal Surfaces. By Marshall Webb, D.D.S. 445

### SELECTED ARTICLES.

ARTICLE III.—Alleged Death from Ether. By Henry J. Bigelow, M. D. .... 451

ARTICLE IV.—Old Way and the New in Dental Practice. By S. Welchens, D. D. S. .... 46[illegible]

ARTICLE V.—Aluminum as a Base for Artificial Teeth. By S. M. Furman .... 469

ARTICLE VI.—Vulcanite Litigation. By S. S. White, D. D. S. .... 471

### EDITORIAL, ETC.

Thirty-fourth Annual Commencement of the Baltimore College of Dental Surgery .... 473

Prof P. H. Austen M. D., D. D. S. .... 473

Letter from W. H. Morgan, D. D. S. .... 473

New Dental Journals .... 474

Convulsions During Dentition .... 475

Webster's Unabridged Dictionary .... 476

### MONTHLY SUMMARY.

Incombustible Paper and Fire-proof Ink .... 477

Use of Sewing Machines .... 478

Counterfeit Diplomas .... 479

Bones from Dissecting Rooms .... 480

Children *versus* Crime .... 480

The Legal Value of Teeth .... 480

